# Antibacterial Characteristics and Activity of Water-Soluble Chitosan Derivatives Prepared by the Maillard Reaction

**DOI:** 10.3390/molecules16108504

**Published:** 2011-10-11

**Authors:** Ying-Chien Chung, Jan-Ying Yeh, Cheng-Fang Tsai

**Affiliations:** 1 Department of Biological Science and Technology, China University of Science and Technology, Taipei 11581, Taiwan; 2 Department of Biotechnology, Asia University, Taichung 41354, Taiwan

**Keywords:** chitosan, water-soluble chitosan, antibacterial activity, Maillard reaction

## Abstract

The antibacterial activity of water-soluble chitosan derivatives prepared by Maillard reactions against *Staphylococcus aureus*, *Listeria monocytogenes*, *Bacillus cereus*, *Escherichia coli*, *Shigella dysenteriae*, and *Salmonella typhimurium* was examined. Relatively high antibacterial activity against various microorganisms was noted for the chitosan-glucosamine derivative as compared to the acid-soluble chitosan. In addition, it was found that the susceptibility of the test organisms to the water-soluble chitosan derivative was higher in deionized water than in saline solution. Metal ions were also found to reduce the antibacterial activity of the water-soluble chitosan derivative on *S. aureus*. The marked increase in glucose level, protein content and lactate dehydrogenase (LDH) activity was observed in the cell supernatant of *S. aureus* exposed to the water-soluble chitosan derivative in deionized water. The results suggest that the water-soluble chitosan produced by Maillard reaction may be a promising commercial substitute for acid-soluble chitosan.

## 1. Introduction

Chitosan is the deacetylated form of chitin, composed of glucosamine, known as 2-amino-2-deoxy-(1→4)-β-D-glucopyranan [1,2]. It is considered to be the most widely distributed biopolymer . It is a cationic, nontoxic, biodegradable, and biocompatible polyelectrolyte, with a p*Ka* of approximately 6.5 [3,4]. It exhibits various potential biological activities, such as antitumor, immunostimulatory, antibacterial and antifungal properties [5,6,7,8]. The exact mechanism of the antimicrobial action of chitosan and its derivatives is still unknown, but several mechanisms have been proposed. Interaction between positively charged chitosan molecules and negatively charged microbial cell membranes leads to the leakage of intracellular constituents [9,10]. Binding of chitosan to DNA triggers inhibition of mRNA synthesis through penetration of the microbial nuclei by chitosan and interfering with the synthesis of mRNA and proteins [11,12]. Kumar and coworkers [13] observed that chitooligomers caused pore formation and permeabilization of the cell wall of *B. cereus*, whereas blockage of nutrient flow due to aggregation of chitooligomers was responsible for the growth inhibition and lysis of *E. coli*. Li and the coworkers [14] indicated that chitosan acetate acted on the bacterial membrane and the antibacterial ativity of chitosan acetate may be due to the disruption or penetration of cell membranes.

Despite its unique antimicrobial properties, the application of chitosan as a food preservative or other uses are limited by its insolubility at neutral or basic pH [14]. Therefore, much effort has been applied to the development of suitable procedures for the preparation of functional derivatives of chitosan and to increase its solubility in water to thus broaden is applications [14,15].

Previously, hydroxyl groups, therefore the introduction of specific monosaccharides (especially glucosamine) into chitosan could be a feasible approach to improve the solubility of chitosan. We did observe that the water-soluble chitosan derivatives effectively enhanced the solubility of chitosan over we had prepared water-soluble chitosan derivatives through Maillard reactions between chitosan and reducing sugars (glucose or glucosamine) as described by Chung and the coworkers [15]. Glucosamine, like chitosan, contains active amino and hydrophilic a relatively wide pH range [15]. These derivatives may overcome the solubility limitations of chitosan at higher pH. It was also found that these chitosan derivatives possessed antioxidative activities [15]. In the present study, we further investigated the antimicrobial activity of the water-soluble chitosan derivatives, and the environmental-related factors, such as pH, chitosan concentration, and salts, associated with the antibacterial activity. In addition, the leakages of intracellular substances, such as glucose, lactate dehydrogenase, and protein, induced by the water-soluble chitosan derivative, were also examined.

## 2. Results and Discussion

### 2.1. Antibacterial Activity of Various Chitosans

The antibacterial activity of two water-soluble chitosans, GN50-5 and GN70-3, prepared by Maillard reactions was assessed using the following six microorganisms: *S. aureus*, *L. monocytogenes*, *B. cereus*, *E. coli*, *Shigella dysenteriae*, and *Salmonella typhimurium.* In general, there is a strong association between antibacterial activity and the cationic amino groups (NH_3_^+^) in chitosan [16]. The water-soluble chitosan derivatives prepared by Maillard reaction was reported to lose partial amino groups [15]. Thus, in this study, the antibacterial activities of the water-soluble chitosan derivatives were examined and compared with the acid-soluble chitosan. Due to the insolubility of the acid-soluble chitosan at pH 7.0 or higher, both the acid-soluble chitosan and water-soluble chitosan derivatives were initially dissolved in 1% acetic acid solution (weak strength solution), then added into nutrient broth to give a final concentration of 250 mg/L. The mixtures were further adjusted with sterile 1 N NaOH solution to a pH of 5.0 or 7.0, respectively.

As shown in Table 1, the growths of six microorganisms were almost completely inhibited by the chitosan-glucosamine derivatives. The water-soluble chitosan derivatives also showed the non-specific antibacterial activity against Gram-positive and Gram-negative microorganisms (Table 1). The water-soluble chitosan derivatives exhibited a significantly higher antibacterial activity than the acid-soluble chitosan at pH 7.0 (Table 1). Furthermore, as shown in Table 1, the antibacterial action of the acid-soluble chitosan was reported to be associated with pH with higher activity at lower pH [17,18]. This may be due to the fact that more amino groups (NH_3_^+^) are formed at pH 5.0 than that at pH 7.0. Besides, due to the poor solubility of the acid-soluble chitosan at neutral pH, white precipitates appeared in nutrient broth while mixing with the acid-soluble chitosan at pH 7.0 [12,15].

**Table 1:** Effect of pH on the antibacterial activity of the water-soluble chitosan derivatives in nutrient broth.

Water-soluble	Antibacterial activity (%) **					
chitosan	*S. aureus*	*L. monocytogenes*	*B. cereus*	*E. coli*	*S. dysenteriae*	*S. tphimurium*
derivative *						
pH 5.0						
GN50-5	100 ± 0	100 ± 0	100 ± 0	100 ± 0	>99 ± 0	>99 ± 0
GN70-3	100 ± 0	100 ± 0	100 ± 0	100 ± 0	100 ± 0	>100 ± 0
Chitosan	>99 ± 0	>99 ± 0	90 ± 0	>99 ± 0	>99 ± 0	>99 ± 0
pH 7.0						
GN50-5	100 ± 0	>99 ± 0	>99 ± 0	>99 ± 0	>99 ± 0	>99 ± 0
GN70-3	100 ± 0	>99 ± 0	>99 ± 0	>99 ± 0	>99 ± 0	>99 ± 0
Chitosan	14 ± 2	11 ± 5	4 ± 2	11 ± 5	5 ± 2	4 ± 1

* Add 250 mg/L concentration of chitosan or water-soluble chitosan derivatives into nutrient broth. ** Each value is expressed as mean ± standard deviation (n = 3).

The solubility of chitosan in solution is critical to its antimicrobial activity, and chitosan solubility is adversely affected by pH > 5.5 [19,20]. Therefore, the lower solubility of acid-soluble chitosan at pH 7.0 may also contribute to the decreased antibacterial activity observed. The higher antibacterial activity was observed for chitosan-glucosamine derivatives, such as GN50-5 and GN70-3, due to the active amino and hydrophilic hydroxyl groups presenting in glucosamine. Thus, the chitosan-glucosamine derivatives soluble in both acidic and basic physiological conditions may be good candidates for the natural bactericidal agents.

### 2.2. Concentration Effect

**Figure 1:**
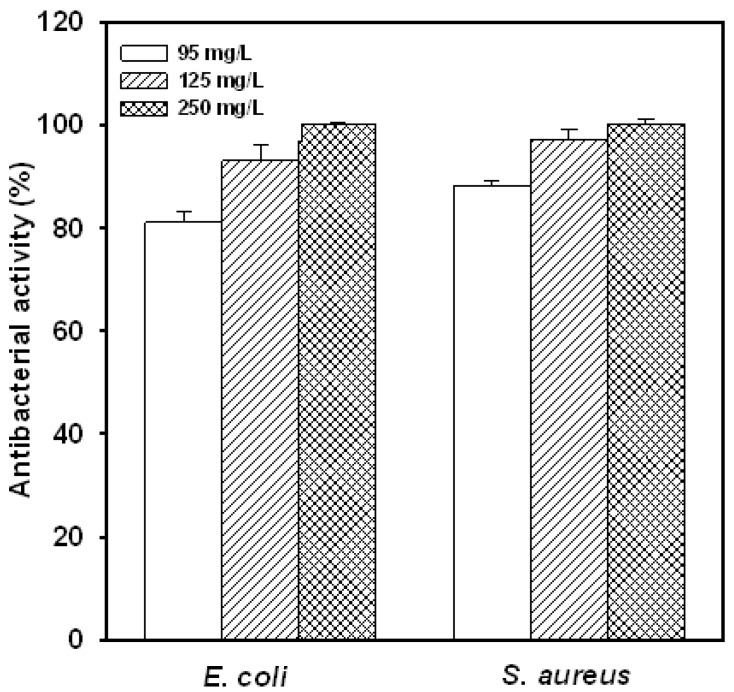
Effect of different concentration of water-soluble chitosan derivative (GN 70-3) on the antibacterial activity of *E. coli and S. aureus* for 6 h at 37 °C in nutrient broth. The initial cell number was 10^7^ CFU/mL. Each value is expressed as mean ± SD (n = 3).

Based on our preliminary results on the yield, solubility, pH stability [15] and antibacterial activity (Table 1), the most promising water-soluble chitosan was GN70-3, thus, this substance was chosen for further investigation in the later experiments. The effect of various concentrations of GN70-3 on antibacterial activity is shown in Figure 1. The antibacterial activities of the water-soluble chitosan on *S. aureus* and *E. coli* in nutrient broth increased markedly as the concentration of the water-soluble chitosan derivative increased. These results were similar to the study that the elevation of the chitosan concentration from 0.5 to 2.5% increased the inhibition on the growths of foodborne pathogens tested [21].

### 2.3. Suspension Medium Influences the Antibacterial Activity of Water-Soluble Chitosan Derivative

**Figure 2:**
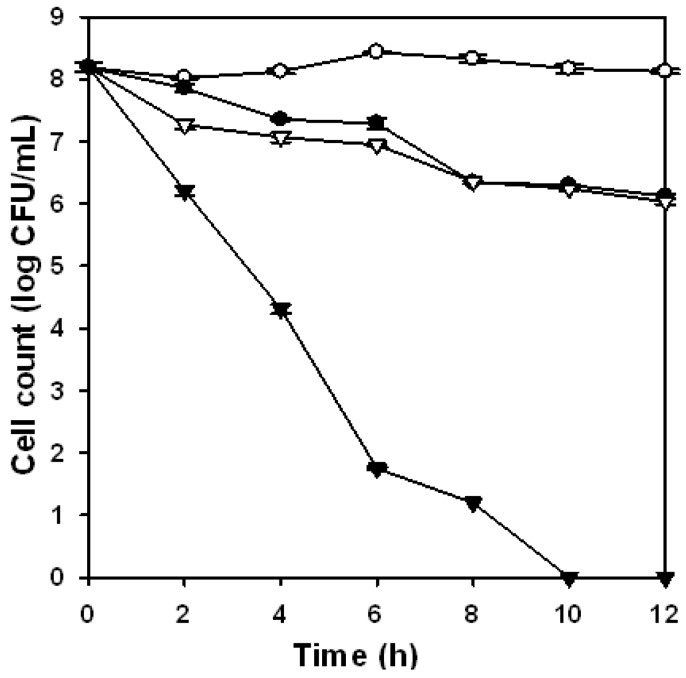
Effect of water-soluble chitosan derivative (GN70-3) at the concentration of 250 mg/L on antibacterial activity of *S. aureus* in deionized water and 0.85% saline solution up to 12 h. *S. aureus* was cultured in either deionized water (▼, ▽) or saline solution (●, ○) at 37 °C. Open symbols (▽, ○) indicates cells without chitosan treatment, while solid symbols (▼, ●) indicated cells with chitosan treatment. Each value is expressed as mean ± SD (n = 3).

The antibacterial activity of the water-soluble chitosan derivative (GN70-3) against *S. aureus* in saline or in deionized water is shown in Figure 2. The antibacterial activity towards *E. coli* was similar to that towards *S. aureus* (data not shown). Regardless of the addition of chitosan derivative in the suspension medium, the viable population of the test microorganism in the same treatment was less in deionized water than that in saline solution. In the same suspension medium, the presence of the water-soluble chitosan derivative, GN70-3, resulted in a less viable population of test microorganism. The viable population of *S. aureus* was reduced from approximately 8.2 log CFU/mL to the non-detectable level after 10 h of incubation in the deionized water containing the water-soluble chitosan derivative. The viable population of test microorganism was reduced slightly in saline solution containing the chitosan derivative, and a viable population of *S. aureus* as high as approximately 6.1 log CFU/mL was observed after 12 h of incubation in saline solution. These results indicate that saline solution markedly reduced the antibacterial activity of GN70-3.

### 2.4. Effect of Metal Ions on the Antibacterial Activity of Water-Soluble Chitosan Derivative

The effect of metal salt addition on the growth of *S. aureus* in nutrient broth with or without water-soluble chitosan derivative (GN70-3) is shown in Table 2. It was found that a reduced final population of ca 4.45 log CFU/mL, with a population reduction of log 3.14 CFU/mL, was noted in the chitosan derivative-containing nutrient broth without metal salt addition compared to that in the respective nutrient broth without chitosan derivative. It appears that the reduced final population observed was the result of the antibacterial action exerted by the chitosan derivative. In combination, the addition of metal ion reduced the antibacterial activity of chitosan derivative and increased the final viable population of *S. aureus* in the chitosan derivative-containing nutrient broth. For example, a final population of 6.28 log CFU/mL was noted in the chitosan derivative-containing nutrient broth supplemented with 15 mM MgCl_2_, although it was less than that in the respective nutrient without chitosan derivative. The antibacterial activity of the chitosan derivative was only ca 39.2% of that exerted in the MgCl_2_-free nutrient broth containing chitosan derivative. Furthermore, as the concentration of metal salt in the chitosan derivative-containing nutrient broth increased, the antibacterial activity of water-soluble chitosan derivative decreased, and resulted in a higher viable population of the test microorganism. The effect of metal salt addition on the growth of *E. coli* was similar to that towards *S. aureus* (data not shown). The decreased antibacterial activity may be due to the chelation of chitosan with the metal ions [17,22]. In addition, the effectiveness of reducing the antibacterial activity of water-soluble chitosan derivative varied with different metal salts added. Among the various metal ions tested, Ba^2+^ ions inhibited the antibacterial activity the most, while the Mg^2+^ ions interfered with it the least. It has been reported that chitosan binds with metal cations through the involvement of -OH and -NH_2_ groups on the glucosamine residues as ligands [23]. Goldberg and the coworkers [24] found that inorganic cations (Na^+^ and Mg^2+^) inhibited the chitosan-mediated adhesion of *E. coli*. Because the -NH_2_ groups are the critical sites for chitosan binding with cells, the chitosan-sodium complex would expect to reduce binding to the cell surface. Young and the coworkers [10] demonstrated that chitosan－induced leakage of *Glycine max* cell was inhibited by divalent cations in the order of Ba^2+^ > Ca^2+^ > Sr^2+^ > Mg^2+^.

**Table 2:** Effect of salt addition on the antibacterial activity of chitosan derivative, GN70-3 (250 mg/L), against S. aureus in nutrient broth.

	Viable population (log CFU/mL) *						Water-soluble		
	Control			GN70-3			chitosan derivative		
							activity (%) **		
	Salt concentration			Salt concentration			Salt concentration		
Salt	0 mM	15 mM	30 mM	0 mM	15 mM	30 mM	0	15	30
							mM	mM	mM
MgCl_2_	7.59 ± 0.06	7.51 ± 0.06B	7.50 ± 0.08B	4.45 ± 0.10	6.28 ± 0.03C	6.53 ± 0.04C	100	39.2	22.9
NaCl	7.59 ± 0.06	7.60 ± 0.04B	7.63 ± 0.02B	4.45 ± 0.10	6.95 ± 0.02A	7.10 ± 0.02A	100	20.7	16.9
CaCl_2_	7.59 ± 0.06	7.55 ± 0.03B	7.57 ± 0.01B	4.45 ± 0.10	6.55 ± 0.04B	6.94 ± 0.05B	100	31.8	20.1
BaCl_2_	7.59 ± 0.06	7.69 ± 0.05A	7.71 ± 0.04A	4.45 ± 0.10	7.09 ± 0.06A	7.23 ± 0.08A	100	19.1	15.3

* Each value is expressed as mean ± standard deviation (n = 3). Means with different letters within a column are significantly different (*p* < 0.05). ** Water-soluble chitosan derivative activity (%) = (difference between log CFU/mL without chitosan derivative and with chitosan derivative in nutrient broth containing same amount of salt)/(difference between log CFU/mL in salt free-nutrient broth without derivative and log CFU/mL in salt free-nutrient containing chitosan derivative).

### 2.5. Cell Leakage Induced by the Water-Soluble Chitosan Derivative

**Figure 3:**
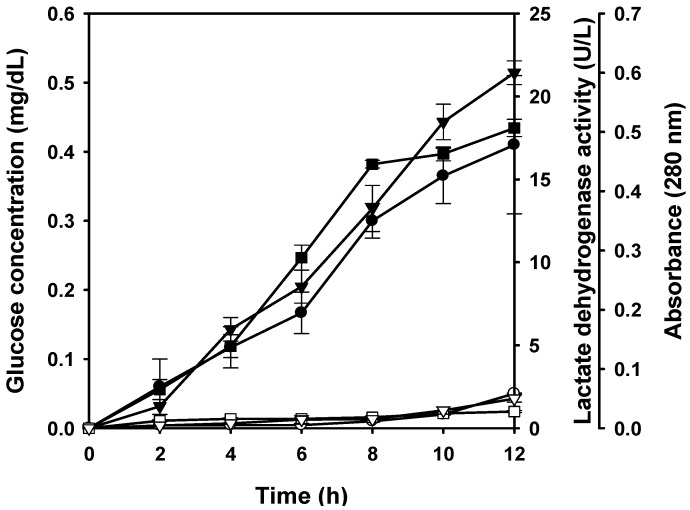
Effect of water-soluble chitosan derivative (GN70-3) at the concentration of 250 mg/L on glucose concentration (○ and ●), lactate dehydrogenase activity (□ and ■) and protein level (▽and ▼) in the extracellular media of *S. aureus* cultured in deionized water at 37 °C up to 12 h. Open symbols (○, ▽, □) indicates cells without chitosan treatment, while solid symbols (●, ▼, ■) indicated cells with chitosan treatment. Each value is expressed as mean ± SD (n = 3).

It is reported that the cell membrane permeability changes as the chitosan molecules interact with the microbial cell surface to cause the leakage of intracellular component and result in cell death [10,23]. In the present study, cell leakage of *S. aureus* induced by water-soluble chitosan derivative (GN70-3) in deionized water was investigated. Figure 3 shows the changes of LDH activity, protein content (absorbance at 280 nm) and glucose concentration in the supernatant of cell suspension after exposure to deionized water with or without water-soluble chitosan derivative. During the 12-h exposure period, the addition of water-soluble chitosan derivative resulted in a higher level of glucose, protein and lactate dehydrogenase (LDH) activity than those observed in the media without water-soluble chitosan derivative-free medium. This indicated that water-soluble chitosan derivative also caused the cell leakage of *S. aureus.* On the other hand, a drastic decline in the viable cell number was observed when the cells of test microorganism were exposed to deionized water containing water-soluble chitosan derivative (Figure 2). In the work by Tsai and Su [23], treatment of chitosan (800 ppm) induced the leakage of glucose and LDH into extracellular media of *E. coli* cells. Yang and the coworkers also indicated that the addition of maltose-chitosan derivative caused the cell leakage in *E. coli* O157:H7 and the glucose level increased continuously during the 12-h period [25]. The reactive amino groups in chitosan could conceivably have the ability to interact with a multitude of anionic groups on the cell surface to alter cell permeability and cause the leakage of intracellular components such as glucose, LDH and protein, and these results may destabilize the cell membrane beyond repair, cause a severe leakage of cell constituents and lead to the cell death. Several authors have proposed that the antimicrobial action of chitosan could be explained by a more direct disturbance of membrane functions [23,26]. In Gram-positive bacteria, the cell membrane is covered by a cell-wall consisting of 30–40 layers of peptidoglycans, which contain GlcNAc, *N*-acetylmuramic acid as well as D- and L-amino acids and teichoic acid [27], to which the positively-charged amino groups of chitosan can bind, result in cell-wall distortion-disruption and expose cell-membrane to osmotic shock and exudation of the cytoplasmic contents [13]. Raafat and the coworkers [28] speculated that binding of chitosan to teichoic acids, coupled with a potential extraction of membrane lipids (predominantly lipoteichoic acid), results in a sequence of events, ultimately leading to bacterial death. Liu and the coworkers [29] showed that chitosan acetate solution increased the permeability of the outer and inner membranes of *E. coli*, and this damage was likely to cause by the electrostatic interaction of NH_3_^+^ groups of chitosan acetate and phosphoryl groups of phospholipids of cell membranes. In short, the results confirmed the water-soluble chitosan derivative to be bactericidal in nature. Although the exact mechanism by which water-soluble chitosan derivatives perform their inhibitory effects on microorganisms remains unknown, the leakage of intracellular constituents may be one of the key factors in cell inactivation.

## 3. Experimental

### 3.1. Chitosan

Chitosan from shrimp shells with 90% *N*-deacetylation as determined by colloid titration [30] was obtained from Lytone Enterprise, Inc. (Taipei, Taiwan). The reagents used were of analytical purity grade.

### 3.2. Preparation of Water-Soluble Chitosan Derivatives

The chitosan derivatives were prepared according to the procedures described by Chung *et al.* [15]. Briefly, chitosan solution 1% (w/v) in 0.2 M glacial acetic acid was prepared. Reducing sugar (glucosamine) was added to the solution and stirred until dissolved. Then the temperature of the reaction vessel was raised to 50 °C or 70 °C for 3 and 5 days. In our previous study, the average yield and solubility reached a maximum on the fifth day at 50 °C, or the third day at 70 °C [15]. Detailed procedures for their preparation and characterization were described in our previous paper [15]. After reaction, the reaction solution was neutralized with 2 M NaOH followed by centrifugation (8,000 × g, 15 min, 15 °C). The supernatant was dialyzed against distilled water by dialysis membrane with molecular weight cut-off 12,000–14,000 (Spectrum Laboratories Inc., Savannah, GA, USA) for 4–6 days and then freeze-dried. The water-soluble chitosan derivatives have been abbreviated as GNt-d, where t is the reaction temperatures and d is reaction times. For example, GN70-3 denoted the reactions with glucosamine at 70 °C for three days. Detailed procedures for their preparation and characterization were described in our previous paper [15].

### 3.3. Assay for Antimicrobial Activity

*Staphylococcus aureus* (CCRC 10777), *Listeria monocytogenes* (CCRC 14845), *Bacillus cereus* (CCRC 10250), *Escherichia coli* (CCRC 10314), *Shigella dysenteriae* (CCRC 13983), and *Salmonella typhimurium* (CCRC 10746) were obtained from the Culture Collection and Research Center (CCRC, Hsinchu, Taiwan). These bacteria were primarily stored in nutrient broth (NB; Difco) containing 50% sterile glycerol at −70 °C. The strains were subcultured twice in nutrient broth and incubated at 37 °C for 12 h. Cells were harvested by centrifugation at 8,000 × g for 15 min, suspended in sterile deionized water or saline solution, and used as the inoculum.

When comparison of the antibacterial activity of acid-soluble and water-soluble chitosan derivatives, each chitosan solution (dissolved in 1% acetic acid) was added to nutrient broth to give a final chitosan concentration of 250 mg/L. The pH of the broth was adjusted to a pH of 5.0 or 7.0 with 1 N NaOH solution before the inoculation of the test organism, and 0.1 mL (about 10^7^ CFU/mL) of each test bacterium was inoculated and incubated at 37 °C with shaking at 120 rpm for 6 h. Then, 0.1 mL of decimal dilutions of samples were spread on nutrient agar plates for colony counting. The antibacterial activity was calculated using [(C − T)/C] × 100%, where C is the colony numbers counted on the control plate and T is that on the tested sample plate [13,31].

On the other hand, various concentrations of chitosan derivative solution (dissolved in distilled water) were prepared to examine the concentration effect. After mixing with nutrient broth, the nutrient broth contained 0–250 mg/L chitosan derivative. It was then inoculated with 0.1 mL of the inoculum of *E. coli* or *S. aureus* and was incubated at 37 °C with shaking at 120 rpm for 6 h.

### 3.4. Effect of Metal Ions

A water-soluble chitosan derivative, GN70-3 (dissolved in distilled water), was added to *S. aureus* cell suspension in nutrient broth containing various concentrations (0, 15, or 30 mM) of MgCl_2_, CaCl_2_, BaCl_2_ or NaCl to give a final concentration of 250 mg/L. The reaction mixtures were incubated at 37 °C with shaking at 120 rpm for 4 h. Surviving cells were counted by spreading on nutrient agar plates at 37 °C for 48 h.

### 3.5. Leakage of Glucose, Lactate Dehydrogenase and Protein from Cells

To examine the effect of water-soluble chitosan derivative on cell leakage and the viability of *S. aureus*, inoculum of the test organism (1 mL) was inoculated into sterile deionized water or saline solution (10 mL) with or without water-soluble chitosan derivative in a culture tube. The mixture, containing 250 mg/L water-soluble chitosan derivative and *S. aureus* cell, was then incubated at 37 °C with shaking (120 rpm) for 12 h. During the pre-determined incubation period, culture tubes were withdrawn for determination of viable cell population, protein and glucose contents and lactate dehydrogenase (LDH) activity.

The cell suspension was centrifuged at 8,000 × g for 15 min, and the supernatant was measured for LDH activity, protein and glucose contents. The glucose content was analyzed by a glucose assay kit (DiaSys Diagnostic Systems GmbH, Holzheim, Germany). A sample or glucose standard (0.0–3.0 mg/dL, 200 μL) was added to reagent (1 mL) containing glucose dehydrogenase. After incubation at 25 °C for 15 min, the absorbance at 334 nm was recorded. The LDH activity was analyzed by an LDH assay kit (Merck, Germany). A 200 μL sample was added to 1 mL reagent containing NADH and incubated at 25 °C. LDH activity was then determined by measuring the rate of decrease of the NADH concentration which was monitored by recording the change of absorbance at 334 nm. The protein concentration was measured by absorbance at 280 nm [10].

### 3.6. Statistical Analysis

The mean values and the standard deviation were from the data of triplicate trials. Mean values were compared by analysis of variance (ANOVA) with Duncan’s multiple range method for comparing groups [32]. A significance level of 5% was adopted for all comparisons.

## 4. Conclusions

The solubility limitation of acid-soluble chitosan was overcome by Maillard reactions with reducing sugars [15]. The water-soluble chitosan derivatives that we obtained exhibited various extents of antibacterial activity against all bacteria tested. At pH 7.0, the antibacterial activity of water-soluble chitosan derivatives was higher than that of acid-soluble chitosan. In addition, suspending medium and metal ion also affect the antibacterial activity of this chitosan derivative. To make the most effective use of this chitosan derivative in future practical applications, such as food preservation, these effects should be noted. In this study, we also observed that the water-soluble chitosan derivative, GN70-3, induced the leakage of cell constituents that may cause the death of *S. aureus* cells. These results suggest that the water-soluble chitosan derivative produced by Maillard reaction is a promising commercial substitute for acid-soluble chitosan. Association of antibacterial activity with water-soluble chitosan derivatives is of value in their utilization as a natural food additive and food preservative, as they are biocompatible and water-soluble for biofunctionalities. In that sense, research and development should be focused on finding novel derivatives of chitosan to increase the antimicrobial activity.
